# The prognostic value of long noncoding RNAs in prostate cancer: a systematic review and meta-analysis

**DOI:** 10.18632/oncotarget.17645

**Published:** 2017-05-07

**Authors:** Weijie Ma, Xi Chen, Lu Ding, Jianhong Ma, Wei Jing, Tian Lan, Haseeb Sattar, Yongchang Wei, Fuling Zhou, Yufeng Yuan

**Affiliations:** ^1^ Department of Hepatobiliary and Pancreatic Surgery, Zhongnan Hospital of Wuhan University, Wuhan, China; ^2^ Department of Clinical Hematology, Zhongnan Hospital of Wuhan University, Wuhan, China; ^3^ Department of Gynaecology and Obstetrics, Zhongnan Hospital of Wuhan University, Wuhan, China; ^4^ Department of Clinical Laboratory Medicine and Center for Gene Diagnosis, Zhongnan Hospital of Wuhan University, Wuhan, China; ^5^ Department of Clinical Pharmacy, Wuhan Union Hospital, Affiliated Hospital, Tongji Medical College, Huazhong University of Science And Technology, Wuhan, China; ^6^ Department of Radiation and Medical Oncology, Zhongnan Hospital of Wuhan University, Wuhan, China

**Keywords:** long non-coding RNA, prognosis, survival, clinicopathology, prostate cancer

## Abstract

The abnormally expressed LncRNAs played irreplaceable roles in the prognosis of prostate cancer (PCa). Therefore, we conducted this systematic review and meta-analysis to summarize the association between the expression of LncRNAs, prognosis and clinicopathology of PCa. 18 eligible studies were recruited into our analysis, including 18 on prognosis and 9 on clinicopathological features. Results indicated that aberrant expression of LncRNAs was significantly associated with biochemical recurrence-free survival (BCR-FS) (HR = 1.55, 95%CI: 1.01–2.37, *P* < 0.05), recurrence free survival (RSF) (HR = 3.07, 95%CI: 1.07–8.86, *P* < 0.05) and progression free survival (PFS) (HR = 2.34, 95%CI: 1.94–2.83, *P* < 0.001) in PCa patients. LncRNAs expression level was correlated with several vital clinical features, like tumor size (HR = 0.52, 95%CI: 0.28–0.95, *P* = 0.03), distance metastasis (HR = 4.55, 95%CI: 2.26–9.15, *P* < 0.0001) and histological grade (HR = 6.23, 95% CI: 3.29–11.82, *P* < 0.00001). Besides, down-regulation of PCAT14 was associated with the prognosis of PCa [over survival (HR = 0.77, 95%CI: 0.63–0.95, *P* = 0.01), BCR-FS (HR = 0.61, 95%CI: 0.48–0.79, *P* = 0.0001), prostate cancer-specific survival (HR = 0.64, 95%CI: 0.48–0.85, *P* = 0.002) and metastasis-free survival (HR = 0.61, 95%CI: 0.50-0.74, *P* < 0.00001)]. And, the increased SChLAP1 expression could imply the worse BCR-FS (HR = 2.54, 95%CI: 1.82-3.56, *P* < 0.00001) and correlate with Gleason score (< 7 vs ≥ 7) (OR = 4.11, 95% CI: 1.94-8.70, *P* = 0.0002). Conclusively, our present work demonstrated that LncRNAs transcription level might be potential prognostic markers in PCa.

## INTRODUCTION

Prostate cancer (PCa) is the most commonly diagnosed cancer and the third leading cause of cancer-related death in men [1]. Histopathological evaluation of biopsy has been set as the golden standard for the diagnosis of PCa, while the drawbacks like infection and bleeding restrained the clinical use [2]. The surveillance for biochemical recurrence (BCR) is one of the vital parameter throughout the treatment of PCa. The low specificity of the widespread diagnostic marker, prostate-specific antigen (PSA), makes it difficult to distinguish indolent or aggressive cancer stages [3]. Without other valuable predictive parameters for early prostate cancer screening, most diagnoses are made in the terminal stage due to the lack of specific and sensitive methods for early prostate cancer screening [4]. Since the high degree of intra-cancer and inter-patient heterogeneity at the molecular level [5], it is an effective to profile the expression of multiple genes to establish the molecular processes occurring in the prostate cancer.

Long non-coding RNAs (LncRNAs) are a class of RNA with transcripts longer than 200 nucleotides and lack functional open reading frames [6]. They can be polyadenylated and may operate in nuclear and/or cytoplasmic fractions. The lack of opening reading frames can either be intergenic, that is located between protein-coding genes, or intragenic, located within an intron of a host protein-coding gene or on the antisense strand [7]. Owing to their biological properties and clinical value in diagnosis, prognosis, and treatment, LncRNAs have been widely investigated. LncRNAs involve in various cell biological processes, like cellular differentiation, proliferation, DNA damage responses and chromosomal imprinting. The abnormal expression of LncRNAs has been reported in various human diseases, including tumors [8]. The lncRNA metastasis associated lung adenocarcinoma transcript 1 (MALAT1) played a vital role in metastasis formation in lung cancer and was a potential therapeutic target [9]. LncRNA-activated by TGF-β (lncRNA-ATB) was significantly up-regulated in hepatocellular carcinoma (HCC) metastases and associated with poor prognosis [10].

In prostate cancer, a well-known example of LncRNAs is the prostate cancer antigen 3 (PCA3; also known as DD3), which overexpresses and promotes invasion and migration in prostate cancer cells by miR-1261 sponging [11]. The level of PCA3 in urine has been used as a diagnostic biomarker for PCa with a sensitivity of 58–82% and a specificity of 56–76% [12–14]. The urinary PCA3 is now widely used for prostate cancer detection and has been approved by the US Food and Drug Administration (FDA) [15]. The expression pattern of lncRNAs also along with coding genes could serve as a prognostic marker. Sun et.al found that MALAT1 was dramatically elevated in human prostate cancer tissues, and its expression was highly associated with Gleason score, tumor stage, PSA level and castration resistance [16]. Besides, decreased expression level of prostate cancer associated transcript-14 (PCAT-14) was prognostic for the metastatic disease and poor survival for patients with prostate cancer [17].

The abnormal expression of lncRNAs could be of prognostic significance. The prognostic value of LncRNAs in PCa has been explored by many studies. The most commonly used methods for detecing prognostic significance include microarray, qRT-PCR, *in situ* hybridization assay (ISH) and available database. However, the inaccuracy and insufficiency caused by the small size and single experiment program might interfere with revealing the real profiles of LncRNAs in PCa. We assumed that he true prognostic value of lncRNAs in PCa could be unravelled through multiple sensitive and reliable detection methods in large scale, multicenter studies. Therefore, we performed the meta-analysis to estimate systematically to explore the potential value of LncRNAs in the prognosis and clinical outcomes in PCa among a relatively larger amount of PCa patients.

## RESULTS

### Study inclusion and characteristics

**Figure 1:**
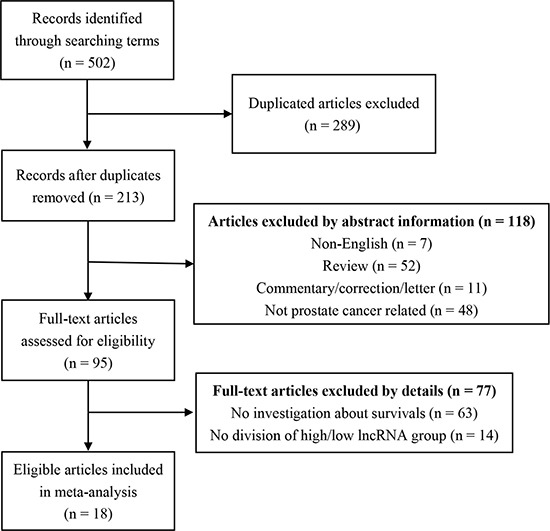
The flow diagram indicated the process of study selection

Initially, we found 502 publications through the internet search from PubMed and the Web of Science. 289 duplicated articles were excluded. After reading the study titles and abstracts, 118 records were removed. Subsequently, the 95 remaining full-text articles were assessed. As a result, a total of 18 articles met the inclusion criteria and were included in the final analysis (Figure 1). Quantitative real-time polymerase chain reaction (qRT-PCR) [18–26]or *in situ* hybridization assay (ISH) [27]was performed to measure the LncRNAs expression. The rest of the studies took advantage of information from several databases which include sequencing data from the cohorts of patients PCa [17, 20, 28–32]. Among these 18 articles, 7 on overall survival (OS) [17, 18, 20, 25, 26, 30, 32], 11 on biochemical recurrence free survival (BCR-FS) [19, 21, 22, 24, 27, 29, 30, 32–34], 2 on recurrence free survival (RFS) [23, 25], 4 on disease free survival (DFS) [18, 28, 29, 33], 3 on metastasis free survival (MFS) [17, 30, 34], 3 on prostate cancer specific survival (PSS) [17, 32], 2 on progression free survival (PFS) [20, 32] (Table 1). Meanwhile, of these 18 studies, 9 articles explored the correlation between LncRNAs and clinicopathological features [17–22, 27, 29, 31] (Table 2).

**Table 1:** Characteristics of studies included in this meta-analysis

Author	Year	LncRNAs	Country	Method	Outcome	Case number(High/Low)	Cut-off	Follow up time
Huang.et al [29]	2017	RP11-108P20.4 /RP11-757G1.6 /RP11-347I19.8 /LINC01123	China	TCGA dataset	BCR-FS & DFS	291(146/145)	median	5000 days
Ghiam.et al [33]	2017	UCA1	Canada	CPC-GENE data & MSKCC database	**CPC-GENE**: BCR-FS; **MSKCC**:DFS	**CPC-GENE**: 209(167/42); **MSKCC**: 130(18/112)	lower 20% and top 80%	10 years
XH Wang.et al [18]	2016	lincRNA-p21	China	qRT-PCR	**Cohort 1** OS & DFS; **Cohort 2** OS & DFS	**Cohort 1**: 81(34/47); **Cohort 2**:66(32/34)	mean	60 months
White.et al [17]	2016	PCAT14	USA	Microarray	MFS & PSS & OS	**MC I**: 545(273/272); **MC II**: 235(118/117); **TJU**: 130(65/65)	median	144 months
Zhang.et al [19]	2016	HCG11	China	qRT-PCR	BCR-FS	138(69/69)	NA	60 months
Shukla.et al [30]	2016	PCAT14	USA	RNA-seq dataset	**JHU**: PSS/MFS/BRC-FS/OS; **Taylor**: BRFS; **TCGA**: MFS	**JHU**: 355(178/177); **Taylor**: 140(NA); **TCGA**: 377(NA)	median	144 months & 150 months
Zheng.et al [20]	2016	CCAT2	China	qRT-PCR	OS & PFS	96(59/37)	median	60 months
Xu.et al [21]	2016	ATB	China	qRT-PCR	BCR-FS	57(25/32)	expression <1.30	100 months
J Wang.et al [22]	2016	LOC400891	China	qRT-PCR	BCR-FS	81(50/31)	two-fold cut-off	60 months
Jiang.et al [23]	2016	MX1-1	China	qRT-PCR	RFS	60(30/30)	NA	60 months
Mehra.et al [27]	2016	SChLAP1	USA	ISH assay	BRC-FS	937(89/848)	score threshold = 100	mean follow-up time 12.8 years
Sakurai.et al [28]	2015	DRAIC	USA	RNA-seq data from MSKCC	DFS	80(69/11)	Z-score = 0.4z	120 months
Na.et al [26]	2015	UCA1	China	qRT-PCR	OS	40(20/20)	median	5 years
Orfanelli.et al [25]	2015	TRPM2-AS	Italy	qRT–PCR	**Sboner**: OS; **Glinksy**: RFS	**Sboner data set**: 199(78/121); **Glinksy data set**:67(28/39)	NA	**Sboner**: 250 months; **Glinksy**: 100 months
Mehra.et al [31]	2014	SChLAP1	USA	ISH assay	RFS	160(33/127)	ISH product score = 100	4000 days
Chakravarty.et al [34]	2014	NEAT1	USA	Affymetrix HuEx microarrays	BCR-FS & MFS	BCR: 216(111/105); MFS: 216(85/131)	NA	70 months
Malik.et al [24]	2014	PCAT29	USA	qRT-PCR	BCR-FS	51(17/34)	high (top 33% of patients) or low (bottom 66% of patients)	>3000 days
Prensner.et al [32]	2013	SChLAP1	USA	Affymetrix exon arrays & qRT-PCR	**Setlur**: OS; **Glinksy**: BCR-FS; **MCTP**: BCR-FS**; Mayo**: BCR-FS & PFS & PSS	**Setlur et al. study**: 357(72/285**); Glinksy et al. study**: 79(16/63); **MCTP** : 65(12/53); **Mayo**: NA	threshold for ‘high’ versus ‘low’ scores = 80%	10 years

BCR-FS = biochemical recurrence-free survival; DFS = disease-free survival; OS = overall survival; MFS = metastasis free survival; PFS = progression free survival; PSS = prostate cancer specific survival; RFS = recurrence free survival; TCGA = The Cancer Genome Atlas dataset; CPC-GENE = Canadian Prostate Cancer Genome Network database; MSKCC = Memorial Sloan Kettering Prostate Cancer database; ISH = in situ hybridization assay; JHU = Johns Hopkins University cohort; Taylor = Taylor.et al cohort; MCI and II = Mayo Clinic I and II cohorts; TJU = Thomas Jefferson University cohort; Sboner = Sboner data set; Glinksy = Glinksy data set; MCT*P* = University of Michigan cohort; Mayo = Mayo Clinic data.

**Table 2:** Association between aberrant levels of lncRNAs and characteristics of patients with PCa

Characteristics	Studies	Case number	Pooled OR (95% CI)	*P*	Heterogeneity		Model	References
					I2	*P*		
Age (≤ 65 vs > 65 years old)	3	468	1.16 [0.45, 2.96]	0.76	19%	0.29	Random	[20, 22, 29]
Lymph node metastasis	8	1971	0.83 [0.48, 1.43]	0.50	64%	0.005	Random	[17–22, 29, 31]
Margin status	5	1478	1.15 [0.66, 2.02]	0.62	71%	0.007	Random	[17, 18, 21, 29, 31]
Preoperative PSA (≤ 10 vs > 10 ng/ml)	3	1011	1.12 [0.23, 5.37]	0.89	89%	0.0001	Random	[17, 18, 21]
SVI	2	1070	2.66 [0.21, 33.15]	0.46	89%	0.003	Random	[17, 31]
ECE/EPE	2	1067	1.30 [0.49, 3.45]	0.60	81%	0.02	Random	[17, 31]
Biochemical recurrence	3	491	2.06 [0.56, 7.57]	0.27	81%	0.005	Random	[19, 21, 31]
Distance Metastasis*	2	177	4.55 [2.26, 9.15]	< 0.0001	0%	0.86	Fixed	[20, 22]
Capsule invasion	2	177	1.36 [0.74, 2.50]	0.32	0%	0.47	Fixed	[20, 22]
Multiple lesions	3	334	0.95 [0.57, 1.58]	0.85	0%	0.82	Fixed	[20, 22, 31]
Tumor diameter (≤ 2.5vs > 2.5 cm)*	2	177	0.52 [0.28, 0.95]	0.03	0%	0.95	Fixed	[20, 22]
Gleason Score (< 7 vs ≥ 7)	8	2678	1.12 [0.54, 2.32]	0.75	82%	< 0.00001	Random	[17, 18, 20–22, 27, 29, 31]
Tumor stage (T2 vs T3-T4)	5	1536	0.88 [0.34, 2.29]	0.79	88%	< 0.00001	Random	[18, 20, 22, 27, 29]
Pathological stage (I + II vs III + IV)	3	1248	2.17 [0.88, 5.37]	0.09	85%	0.001	Random	[19, 21, 27]
Histological grade (II vs III + IV)*	2	177	6.23 [3.29, 11.82]	< 0.00001	0%	0.81	Fixed	[20, 22]

SVI = seminal vesical involvement; ECE = extra capsular extension; EPE = extra prostatic extension. “*” means *P* < 0.05.

### Prognostic value for PCa

**Figure 2:**
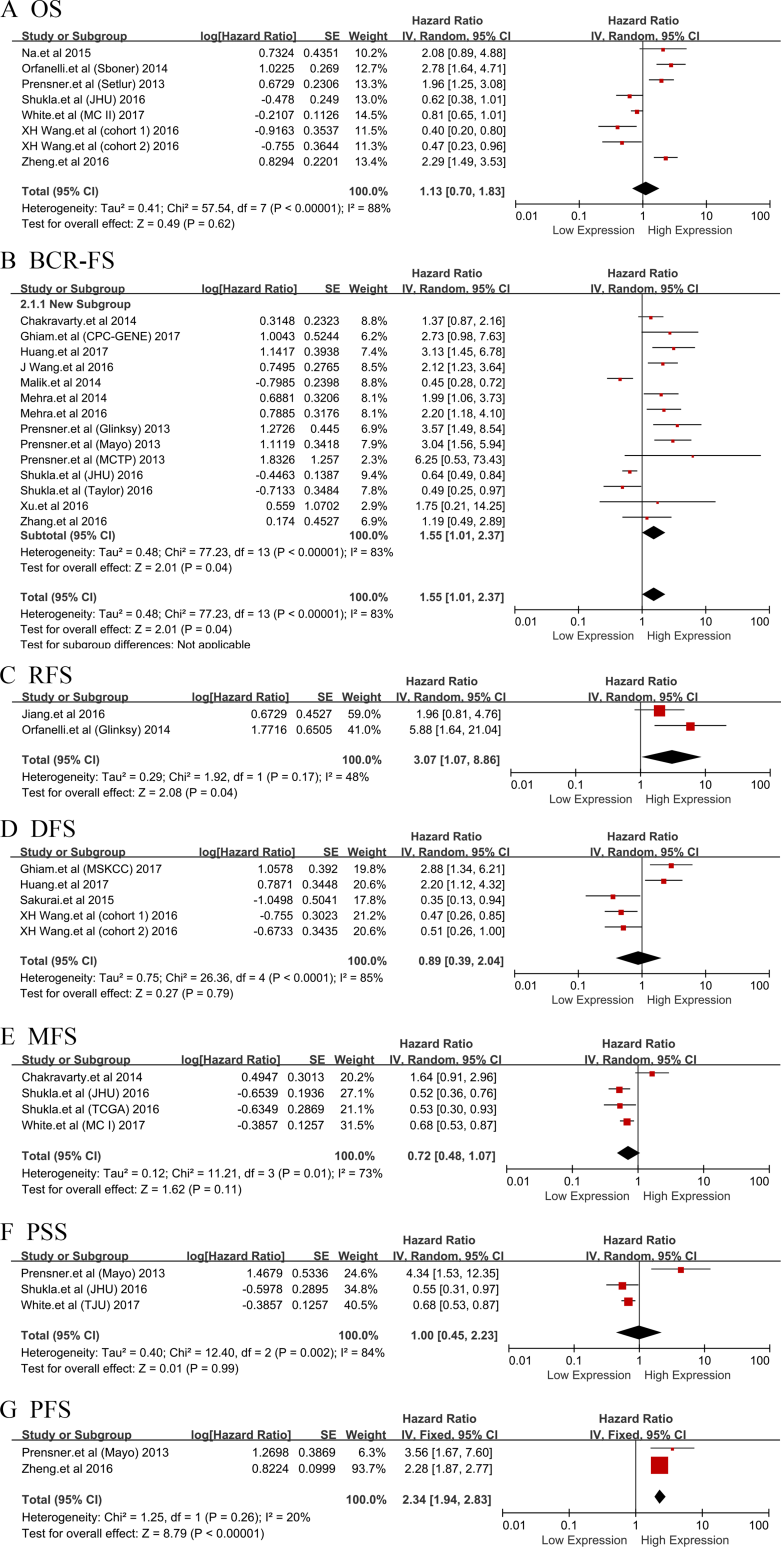
Forest plot of studies evaluating hazard ratios of LncRNAs expression and prognosis in PCa The point estimate is bounded by a 95% confidence interval, and the perpendicular line represents no increased risk for the outcome. OS: overall survival; BCR-FS: biochemical recurrence-free survival; RFS: recurrence free survival; DFS: disease-free survival; MFS: metastasis free survival; PSS: prostate cancer specific survival; PFS: progression free survival.

We conducted the correlation between LncRNAs expression level and survivals among 5242 patients diagnosed with PCa from 18 included studies. 17 different aberrant LncRNAs were correlated with the prognosis of PCa patients. From the frost plots, the up-regulation of RP11-347I19.8/LINC01123 [29], UCA1 [33], HCG11 [19], CCAT2 [20], ATB [21], LOC400891 [22], MX1-1 [23] , SChLAP1 [27, 31, 32] , NEAT1 [34] and TRPM2-AS [25] were associated with poor prognosis. While, the down-regulation of RP11-108P20.4 /RP11-757G1.6 [29], lincRNA-p21 [18], PCAT14 [17, 30], DRAIC [28] and PCAT29 [24] implied the poor prognosis (Figure 2).

**Figure 3:**
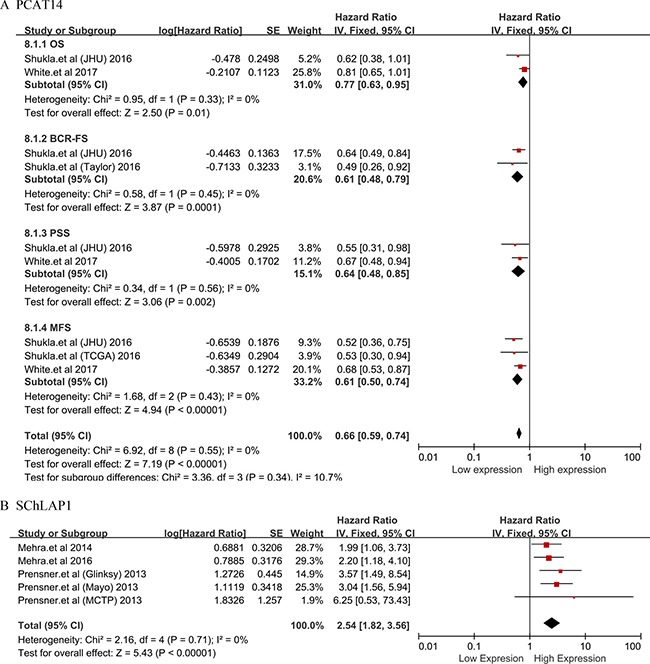
Forest plots of studies evaluating hazard ratios of PCAT14 and SChLAP1 with the prognosis of PCa (**A**)PCAT14; (**B**) SChLAP1, biochemical recurrence-free survival (BCR-FS).

Subsequently, PCAT14 and SChLAP1 which were performed no less than two studies were included into meta-analysis on the relationship between the expression level and the prognosis of patients with PCa, respectively. We found that all the heterogeneities were not significant (I^2^ = 0.0%, *P* > 0.05) (Figure 3). Thus, we applied the fixed effects model to conduct the analysis. We found that the down-regulated PCAT14 level was associated with a poor OS (HR = 0.77, 95% CI = 0.63 to 0.95, *P* = 0.01), BCR-FS (HR = 0.61, 95% CI = 0.48 to 0.79, *P* = 0.0001), PSS (HR = 0.64, 95% CI = 0.48 to 0.85, *P* = 0.002) and MFS (HR = 0.61, 95% CI = 0.50 to 0.74, *P* < 0.00001) (Figure 3A). While, the increased SChLAP1 expression could implied the worse BCR-FS (HR = 2.54, 95% CI = 1.82 to 3.56, *P* < 0.00001) (Figure 3B).

### The correlation between LncRNAs and clinicopathological features

**Figure 4:**
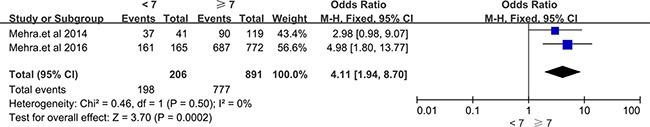
Forest plots of studies evaluating odds ratios (ORs) of up-regulated SChLAP1 expression and Gleason Score(< 7 vs ≥ 7) of PCa patients

A total of 11 LncRNAs described in 9 included articles showed the association with clinicopathological features of prostate cancer. RP11-108P20.4 /RP11-757G1.6 [29], lincRNA-p21 [18], PCAT14 [17] were reported decreased expression in PCa, while RP11-347I19.8/LINC01123 [29], HCG11 [19], CCAT2 [20], ATB [21], LOC400891 [22], SChLAP1 [27, 31] were overexpressed in PCa. Through the meta-analysis, we found that the aberrant expression of LncRNAs were significantly correlated with distance metastasis (OR = 4.55, 95% CI = 2.26 to 9.15, *P* < 0.0001, fixed effect), tumor diameter (OR = 0.52, 95% CI = 0.28 to 0.95, *P* = 0.03, fixed effect), histological grade (OR = 6.23, 95% CI = 3.29 to 11.82, *P* < 0.00001, fixed effect). Unfortunately, there were no statistical significance in the correlation between LncRNAs expression level and the clinical data like gender, lymph node metastasis, preoperative PSA and so on (see details in Table 2). Two studies revealed that up-regulated SChLAP1 was significantly related to the Gleason score [27, 31]. Statistical significance emerged when we performed meta-analysis among these two articles (Gleason score < 7 vs ≥ 7, OR = 4.11, 95% CI = 1.94 to 8.70, *P* = 0.0002, fixed model) (Figure 4).

### Publication bias and sensitivity analysis

We applied Begg's test to estimate the publication bias among these studies. All the Begg's tests in our analysis showed no publication bias, due to the value of *P* > 0.05, respectively. The sensitivity analysis which was performed by Stata11.0 software evaluated the stability of our results. We found that no individual study significantly interfered with the overall results which demonstrated the credibility of the present meta-analysis (Supplementary Figures 1–4).

## DISCUSSION

Long non-coding RNA contained more than 200 nucleotides constitutes a great proportion of non-coding transcripts [35]. Many LncRNAs exhibited cell-type specific expression and located in specific subcellular compartments [36, 37]. LncRNAs could function as a role of molecular scaffolds for targeting gene regulatory proteins/complexes to specific genomic loci [7]. So, they could influence the expression of target proteins of neighboring protein-coding genes, regulate the distal transcriptional elements and modulate the activity of protein-binding partners [38–40]. Furthermore, LncRNAs could act as a suppressor or activator of gene expression. The increase or decrease of a number of LncRNAs contribute to oncogenesis by influencing many cellular processes [41].

The aberrant expression of LncRNAs is related to the development and progression of prostate cancer through affecting tumor cell proliferation, metastasis, self-renewal, survival, and apoptosis by either transcriptional or post-transcriptional regulation [42]. Several PCa-specific LncRNAs have been reported, and some are associated with distinct subtypes of the disease. In prostate cancer, the up-regulated prostate cancer antigen 3 (PCA3; also known as DD3), is already available as a diagnostic test in urine [43, 44]. It has indicated that the overexpressed PCA3 could modulate prostate cancer cells survival by altering androgen receptor (AR) signaling [45]. Besides, the lately study elaborated that PCGEM1 and PRNCR1, bound successively to the androgen receptor and strongly enhanced both ligand-dependent and ligand-independent androgen-receptor-mediated gene activation programs and proliferation in prostate cancer cells [46]. Apart from Gleason score, the increased expression of SChLAP1 was validated as a significantly prognostic biomarker for metastatic prostate cancer increased with prostate cancer progression and predicted the poor clinical outcome in patients with localized prostate cancer following radical prostatectomy and patients with lethal prostate cancer [27, 31, 47] . The upregulation of SChLAP1 in PCa patients could lead to poor outcomes, including metastasis and prostate cancer-specific mortality, by antagonizing the tumor-suppressive functions of the SWI/SNF complex [32]. While, a novel prostate cancer and lineage-specific LncRNA PCAT14, which is transcriptionally regulated by AR, is overexpressed in low grade disease and lack of PCAT14 predicts for disease aggressiveness and recurrence in PCa [30].

On the purpose of detecting the prognostic value of LncRNAs in PCa, we performed this comprehensive systematic review and meta-analysis of the current literature which is the first systematical analysis of the relationship between LncRNAs expression level with prognosis and clinical features of PCa. Our results demonstrated that the high expression of 11 LncRNAs was related with poor prognosis, so was the low expression of 6 LncRNAs. PCAT14 and SChLAP1 were reported by no less than two studies, thus, subsequently, we conducted meta-analysis for prognostic value of these two LncRNAs in PCa, respectively. We found that the decreased PCAT14 expression could predict poor OS, BCR-FS, PSS and MFS in PCa patients. While the overexpressed SChLAP1 among PCa patients had worse BCR-FS. Regarding the relationship with clinicopathological features, the increased expression level of CCAT2 and LOC400891 could be the identifiers of an existence of distance metastasis, tumor diameter (≤ 2.5 vs > 2.5 cm) and histological grade (II vs III + IV) for PCa. The level of SChLAP1 existed a significant difference between the group with Gleason score < 7 and ≥ 7. The non-significant correlation between LncRNAs and other characters might be caused by the insufficient studies for each LncRNA.

However, several limitations existed in our analysis should be considered. The included studies in our meta-analysis weren't sufficient with limited sample size and all were English researches. No study with negative results was included in our analysis which could amplify the relation between LncRNAs and clinical values of PCa. Studies contained diverse LncRNAs used different follow-up endpoints. Besides, the cut-off value distinguished high or low levels of LncRNAs differed among these studies.

In conclusion, our study was the first meta-analysis to evaluate the clinical value of expression level of LncRNAs in prostate cancer. Despite the limitation, we demonstrated that transcription level was correlated with prognosis of PCa and several vital clinical characters. However, further comprehensive and large-scale research should be performed to confirm our findings.

## MATERIALS AND METHODS

### Literature search strategy and study eligibility criteria

We searched databases like PubMed and Web of Science for studies published in English up to February 17, 2017. The following keywords were used “Long noncoding RNA” or “Long intergenic non-coding RNA” or “lncRNA” or “LincRNA” and “prostate cancer” or “PCa” with the limit to human.

### Criteria of eligibility

The inclusion criteria for our meta-analysis were: (1) articles published as a full paper in English; (2) all patients were diagnosed with PCa; (3) LncRNAs expression levels were measured in PCa tissues; (4) the association of LncRNAs with survivals (OS/ BCR-FS/ RFS/ DFS/ MFS/ PSS/ PFS) was detected; (5) correlation between LncRNAs and clinicopathological features was performed at least two parameters; (6) studies provided sufficient information to estimate hazard ratios (HR) and 95% confidence interval (95% CI). Studies which failed to provide enough data were excluded from this meta-analysis. Only the latest or most complete data were chosen when we dealt with duplicated publications.

### Data extraction

The usable data were extracted independently by two reviewers (Ma WJ and Jing W). Any disagreements between the three reviewers were resolved by consensus involving other two reviewers (Chen X, Ding L and Ma JH). The reviewers screened the name of first author, year of publication, country, the type of LncRNAs, a method for detection of LncRNAs, cut-off value and the follow-up time, clinicopathological parameters and the HRs with 95% CIs for survival analysis.

### Statistical analysis

The HRs and 95% CI were used to evaluate the association between lncRNAs and prognosis. A provided HR > 1 implied a poor survival for the high expressed lncRNAs group. On the contrary, HR < 1 meant a worse survival for the group with decreased lncRNAs expression level. We extracted HR according to the following two methods: (1) The HRs and 95% CI were obtained directly from the publication; (2) We calculated the HRs and 95%CI by extracting several survival rates from the Kaplan–Meier survival curves using Engauge Digitizer version 4.1. The second method may generate errors by variation. Meanwhile, Aiming to investigate the relationship between the expression of lncRNAs and clinicopathologic characteristics, the ORs and 95% CI were used.

All analyses were performed using the STATA software version 11.0 and Cochrane Collaboration Review Manager Version 5.2. To investigate the heterogeneity among studies, I^2^ statistics and chi-square *Q* test were used. When I^2^ value more than 50% or a *P*-value less than 0.05 for *Q* test, the heterogeneity was regarded as significant. Fixed-effects model was used when there was no significant heterogeneity between studies. Otherwise, the random-effects model was used. We also performed sensitivity analyses to test the effect of each study on pooled results. Begg's test was applied for assessing publication bias. Statistical significance was defined when a *P* < 0.05.

## SUPPLEMENTARY MATERIALS FIGURES



## References

[1] Siegel RL, Miller KD, Jemal A (2017). Cancer Statistics, 2017. CA Cancer J Clin.

[2] Loeb S, Vellekoop A, Ahmed HU, Catto J, Emberton M, Nam R, Rosario DJ, Scattoni V, Lotan Y (2013). Systematic review of complications of prostate biopsy. Eur Urol.

[3] Prensner JR, Rubin MA, Wei JT, Chinnaiyan AM (2012). Beyond PSA: the next generation of prostate cancer biomarkers. Sci Transl Med.

[4] Wilt TJ, Brawer MK, Jones KM, Barry MJ, Aronson WJ, Fox S, Gingrich JR, Wei JT, Gilhooly P, Grob BM, Nsouli I, Iyer P, Cartagena R (2012). Radical prostatectomy versus observation for localized prostate cancer. N Engl J Med.

[5] Fraser M, Berlin A, Bristow RG, van der Kwast T (2015). Genomic, pathological, and clinical heterogeneity as drivers of personalized medicine in prostate cancer. Urol Oncol.

[6] Guttman M, Rinn JL (2012). Modular regulatory principles of large non-coding RNAs. Nature.

[7] Walsh AL, Tuzova AV, Bolton EM, Lynch TH, Perry AS (2014). Long noncoding RNAs and prostate carcinogenesis: the missing ‘linc’?. Trends Mol Med.

[8] Brunner AL, Beck AH, Edris B, Sweeney RT, Zhu SX, Li R, Montgomery K, Varma S, Gilks T, Guo X, Foley JW, Witten DM, Giacomini CP (2012). Transcriptional profiling of long non-coding RNAs and novel transcribed regions across a diverse panel of archived human cancers. Genome Biol.

[9] Gutschner T, Hammerle M, Eissmann M, Hsu J, Kim Y, Hung G, Revenko A, Arun G, Stentrup M, Gross M, Zornig M, MacLeod AR, Spector DL, Diederichs S (2013). The noncoding RNA MALAT1 is a critical regulator of the metastasis phenotype of lung cancer cells. Cancer Res.

[10] Yuan JH, Yang F, Wang F, Ma JZ, Guo YJ, Tao QF, Liu F, Pan W, Wang TT, Zhou CC, Wang SB, Wang YZ, Yang Y (2014). A long noncoding RNA activated by TGF-beta promotes the invasion-metastasis cascade in hepatocellular carcinoma. Cancer Cell.

[11] He JH, Li BX, Han ZP, Zou MX, Wang L, Lv YB, Zhou JB, Cao MR, Li YG, Zhang JZ (2016). Snail-activated long non-coding RNA PCA3 up-regulates PRKD3 expression by miR-1261 sponging, thereby promotes invasion and migration of prostate cancer cells. Tumour Biol.

[12] Tinzl M, Marberger M, Horvath S, Chypre C (2004). DD3PCA3 RNA analysis in urine--a new perspective for detecting prostate cancer. Eur Urol.

[13] van Gils MP, Hessels D, van Hooij O, Jannink SA, Peelen WP, Hanssen SL, Witjes JA, Cornel EB, Karthaus HF, Smits GA, Dijkman GA, Mulders PF, Schalken JA (2007). The time-resolved fluorescence-based PCA3 test on urinary sediments after digital rectal examination; a Dutch multicenter validation of the diagnostic performance. Clin Cancer Res.

[14] Marks LS, Fradet Y, Deras IL, Blase A, Mathis J, Aubin SM, Cancio AT, Desaulniers M, Ellis WJ, Rittenhouse H, Groskopf J (2007). PCA3 molecular urine assay for prostate cancer in men undergoing repeat biopsy. Urology.

[15] Groskopf J, Aubin SM, Deras IL, Blase A, Bodrug S, Clark C, Brentano S, Mathis J, Pham J, Meyer T, Cass M, Hodge P, Macairan ML (2006). APTIMA PCA3 molecular urine test: development of a method to aid in the diagnosis of prostate cancer. Clin Chem.

[16] Ren S, Liu Y, Xu W, Sun Y, Lu J, Wang F, Wei M, Shen J, Hou J, Gao X, Xu C, Huang J, Zhao Y, Sun Y (2013). Long noncoding RNA MALAT-1 is a new potential therapeutic target for castration resistant prostate cancer. J Urol.

[17] White NM, Zhao SG, Zhang J, Rozycki EB, Dang HX, McFadden SD, Eteleeb AM, Alshalalfa M, Vergara IA, Erho N, Arbeit JM, Karnes RJ, Den RB (2017). Multi-institutional Analysis Shows that Low PCAT-14 Expression Associates with Poor Outcomes in Prostate Cancer. Eur Urol.

[18] Wang X, Ruan Y, Wang X, Zhao W, Jiang Q, Jiang C, Zhao Y, Xu Y, Sun F, Zhu Y, Xia S, Xu D (2017). Long intragenic non-coding RNA lincRNA-p21 suppresses development of human prostate cancer. Cell Prolif.

[19] Zhang Y, Zhang P, Wan X, Su X, Kong Z, Zhai Q, Xiang X, Li L, Li Y (2016). Downregulation of long non-coding RNA HCG11 predicts a poor prognosis in prostate cancer. Biomed Pharmacother.

[20] Zheng J, Zhao S, He X, Zheng Z, Bai W, Duan Y, Cheng S, Wang J, Liu X, Zhang G (2016). The up-regulation of long non-coding RNA CCAT2 indicates a poor prognosis for prostate cancer and promotes metastasis by affecting epithelial-mesenchymal transition. Biochem Biophys Res Commun.

[21] Xu S, Yi XM, Tang CP, Ge JP, Zhang ZY, Zhou WQ (2016). Long non-coding RNA ATB promotes growth and epithelial-mesenchymal transition and predicts poor prognosis in human prostate carcinoma. Oncol Rep.

[22] Wang J, Cheng G, Li X, Pan Y, Qin C, Yang H, Hua L, Wang Z (2016). Overexpression of long non-coding RNA LOC400891 promotes tumor progression and poor prognosis in prostate cancer. Tumour Biol.

[23] Jiang CY, Gao Y, Wang XJ, Ruan Y, Bei XY, Wang XH, Jing YF, Zhao W, Jiang Q, Li J, Han BM, Xia SJ, Zhao FJ (2016). Long non-coding RNA lnc-MX1-1 is associated with poor clinical features and promotes cellular proliferation and invasiveness in prostate cancer. Biochem Biophys Res Commun.

[24] Malik R, Patel L, Prensner JR, Shi Y, Iyer MK, Subramaniyan S, Carley A, Niknafs YS, Sahu A, Han S, Ma T, Liu M, Asangani IA (2014). The lncRNA PCAT29 inhibits oncogenic phenotypes in prostate cancer. Mol Cancer Res.

[25] Orfanelli U, Jachetti E, Chiacchiera F, Grioni M, Brambilla P, Briganti A, Freschi M, Martinelli-Boneschi F, Doglioni C, Montorsi F, Bellone M, Casari G, Pasini D, Lavorgna G (2015). Antisense transcription at the TRPM2 locus as a novel prognostic marker and therapeutic target in prostate cancer. Oncogene.

[26] Na XY, Liu ZY, Ren PP, Yu R, Shang XS (2015). Long non-coding RNA UCA1 contributes to the progression of prostate cancer and regulates proliferation through KLF4-KRT6/13 signaling pathway. Int J Clin Exp Med.

[27] Mehra R, Udager AM, Ahearn TU, Cao X, Feng FY, Loda M, Petimar JS, Kantoff P, Mucci LA, Chinnaiyan AM (2016). Overexpression of the Long Non-coding RNA SChLAP1 Independently Predicts Lethal Prostate Cancer. Eur Urol.

[28] Sakurai K, Reon BJ, Anaya J, Dutta A (2015). The lncRNA DRAIC/PCAT29 Locus Constitutes a Tumor-Suppressive Nexus. Mol Cancer Res.

[29] Huang TB, Dong CP, Zhou GC, Lu SM, Luan Y, Gu X, Liu L, Ding XF (2017). A potential panel of four-long noncoding RNA signature in prostate cancer predicts biochemical recurrence-free survival and disease-free survival. Int Urol Nephrol.

[30] Shukla S, Zhang X, Niknafs YS, Xiao L, Mehra R, Cieslik M, Ross A, Schaeffer E, Malik B, Guo S, Freier SM, Bui HH, Siddiqui J (2016). Identification and Validation of PCAT14 as Prognostic Biomarker in Prostate Cancer. Neoplasia.

[31] Mehra R, Shi Y, Udager AM, Prensner JR, Sahu A, Iyer MK, Siddiqui J, Cao X, Wei J, Jiang H, Feng FY, Chinnaiyan AM (2014). A novel RNA in situ hybridization assay for the long noncoding RNA SChLAP1 predicts poor clinical outcome after radical prostatectomy in clinically localized prostate cancer. Neoplasia.

[32] Prensner JR, Iyer MK, Sahu A, Asangani IA, Cao Q, Patel L, Vergara IA, Davicioni E, Erho N, Ghadessi M, Jenkins RB, Triche TJ, Malik R (2013). The long noncoding RNA SChLAP1 promotes aggressive prostate cancer and antagonizes the SWI/SNF complex. Nat Genet.

[33] Fotouhi Ghiam A, Taeb S, Huang X, Huang V, Ray J, Scarcello S, Hoey C, Jahangiri S, Fokas E, Loblaw A, Bristow RG, Vesprini D, Boutros P, Liu SK (2017). Long non-coding RNA urothelial carcinoma associated 1 (UCA1) mediates radiation response in prostate cancer. Oncotarget.

[34] Chakravarty D, Sboner A, Nair SS, Giannopoulou E, Li R, Hennig S, Mosquera JM, Pauwels J, Park K, Kossai M, MacDonald TY, Fontugne J, Erho N (2014). The oestrogen receptor alpha-regulated lncRNA NEAT1 is a critical modulator of prostate cancer. Nat Commun.

[35] St Laurent G, Shtokalo D, Tackett MR, Yang Z, Eremina T, Wahlestedt C, Urcuqui-Inchima S, Seilheimer B, McCaffrey TA, Kapranov P (2012). Intronic RNAs constitute the major fraction of the non-coding RNA in mammalian cells. BMC Genomics.

[36] Cesana M, Cacchiarelli D, Legnini I, Santini T, Sthandier O, Chinappi M, Tramontano A, Bozzoni I (2011). A long noncoding RNA controls muscle differentiation by functioning as a competing endogenous RNA. Cell.

[37] Lee B, Mazar J, Aftab MN, Qi F, Shelley J, Li JL, Govindarajan S, Valerio F, Rivera I, Thurn T, Tran TA, Kameh D, Patel V, Perera RJ (2014). Long noncoding RNAs as putative biomarkers for prostate cancer detection. J Mol Diagn.

[38] Moran VA, Perera RJ, Khalil AM (2012). Emerging functional and mechanistic paradigms of mammalian long non-coding RNAs. Nucleic Acids Res.

[39] Lee JT (2012). Epigenetic regulation by long noncoding RNAs. Science.

[40] Mariner PD, Walters RD, Espinoza CA, Drullinger LF, Wagner SD, Kugel JF, Goodrich JA (2008). Human Alu RNA is a modular transacting repressor of mRNA transcription during heat shock. Mol Cell.

[41] Ma L, Bajic VB, Zhang Z (2013). On the classification of long non-coding RNAs. RNA Biol.

[42] Su YJ, Yu J, Huang YQ, Yang J (2015). Circulating Long Noncoding RNA as a Potential Target for Prostate Cancer. Int J Mol Sci.

[43] Ploussard G, de la Taille A (2010). Urine biomarkers in prostate cancer. Nat Rev Urol.

[44] Ploussard G, Haese A, Van Poppel H, Marberger M, Stenzl A, Mulders PF, Huland H, Bastien L, Abbou CC, Remzi M, Tinzl M, Feyerabend S, Stillebroer AB (2010). The prostate cancer gene 3 (PCA3) urine test in men with previous negative biopsies: does free-to-total prostate-specific antigen ratio influence the performance of the PCA3 score in predicting positive biopsies?. BJU Int.

[45] Ferreira LB, Palumbo A, de Mello KD, Sternberg C, Caetano MS, de Oliveira FL, Neves AF, Nasciutti LE, Goulart LR, Gimba ER (2012). PCA3 noncoding RNA is involved in the control of prostate-cancer cell survival and modulates androgen receptor signaling. BMC Cancer.

[46] Yang L, Lin C, Jin C, Yang JC, Tanasa B, Li W, Merkurjev D, Ohgi KA, Meng D, Zhang J, Evans CP, Rosenfeld MG (2013). lncRNA-dependent mechanisms of androgen-receptor-regulated gene activation programs. Nature.

[47] Prensner JR, Zhao S, Erho N, Schipper M, Iyer MK, Dhanasekaran SM, Magi-Galluzzi C, Mehra R, Sahu A, Siddiqui J, Davicioni E, Den RB, Dicker AP (2014). RNA biomarkers associated with metastatic progression in prostate cancer: a multi-institutional high-throughput analysis of SChLAP1. Lancet Oncol.

