# Supplementary material for: The prognostic value of long noncoding RNAs in prostate cancer: a systematic review and meta-analysis
Source: Oncotarget. 2017 May 7;8(34):57755–65. doi: 10.18632/oncotarget.17645 (PMC5593681; doi:10.18632/oncotarget.17645)
Supplement: Supplementary file 1 [file oncotarget-08-57755-s001.pdf]

The prognostic value of long noncoding RNAs in prostate cancer: A systematic review and meta-analysis

Supplementary Materials

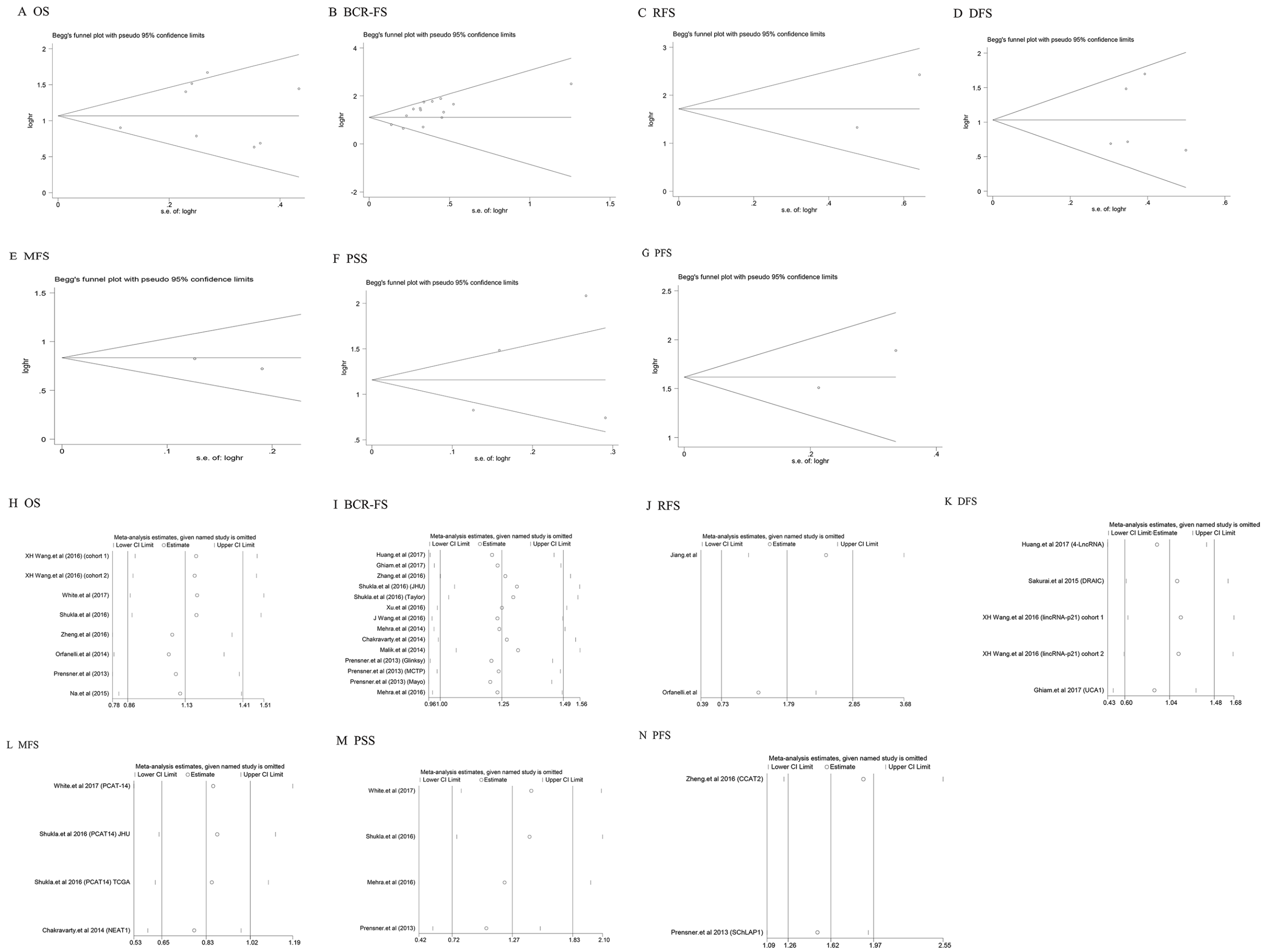

Supplementary Figure 1: Begg's test for publication bias and sensitivity analyses of the studies for the prognostic value of LncRNAs in PCa.

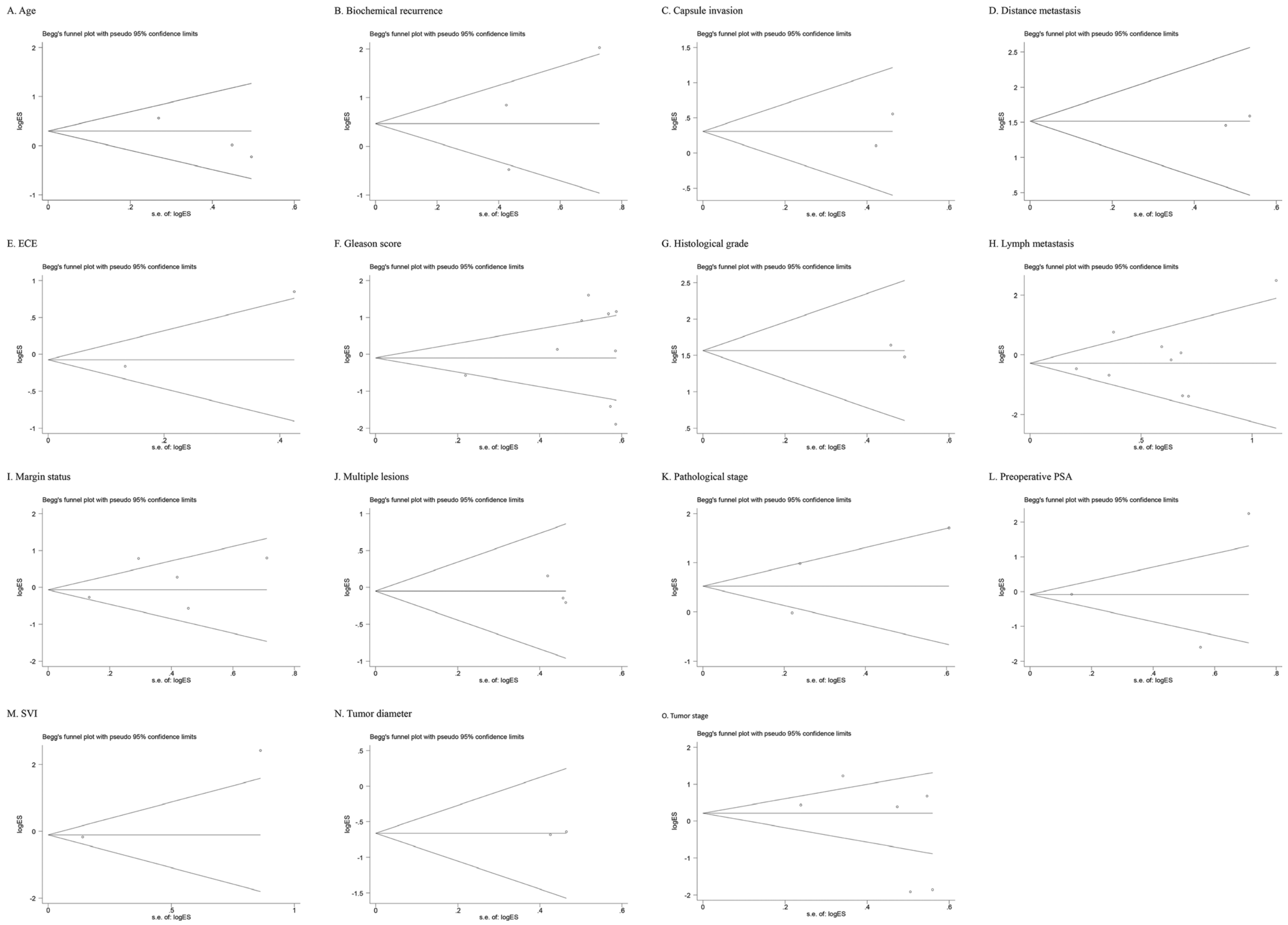

Supplementary Figure 2: Begg's test for publication bias for the correlation between expression level of LncRNAs and clinical features in PCa.

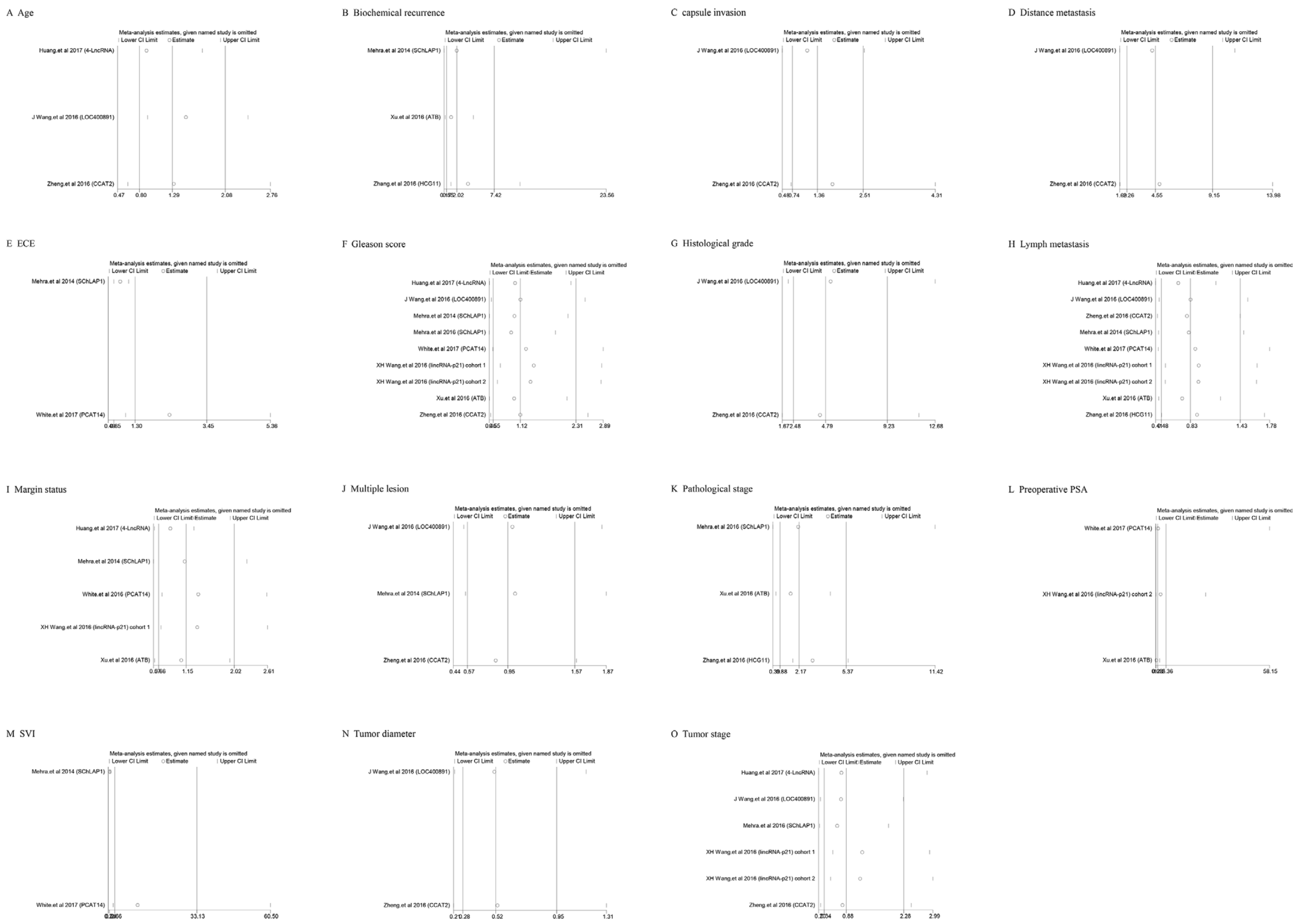

Supplementary Figure 3: Sensitivity analyses of the studies for the correlation between expression level LncRNAs and clinical features in PCa.

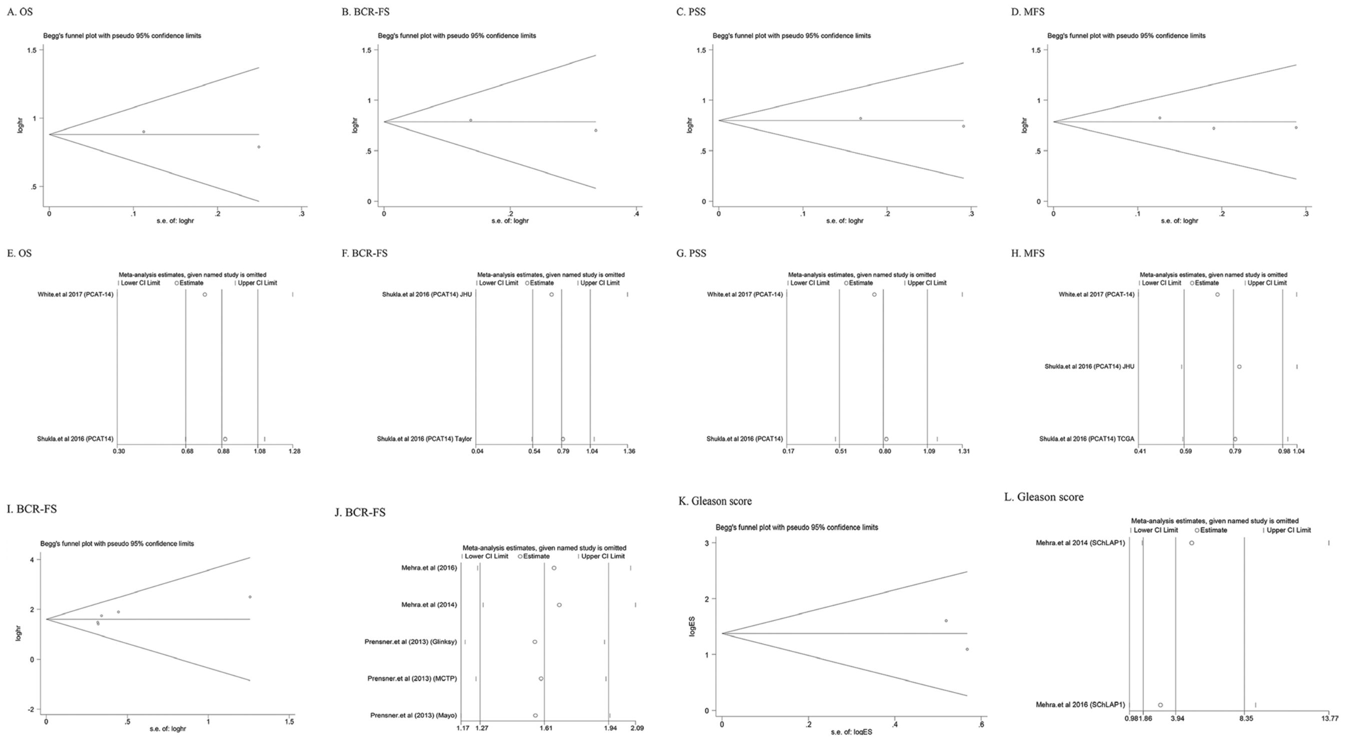

Supplementary Figure 4: Begg's test for publication bias and sensitivity analyses of the studies for the prognostic value of PCAT14 in PCa and the relation of SChLAP1 with BCR-FS and Gleason score (< 7 vs ≥ 7) in PCa. A - J. PCAT14; I - L. SChLAP1.
